# Editorial: Emerging materials and structures for future renewable energy conversion and large-scale storage technology

**DOI:** 10.3389/fchem.2025.1732798

**Published:** 2025-11-13

**Authors:** Wenxiu Que, Xingtian Yin, Yawei Yang, Fengyu Shen

**Affiliations:** 1 Electronic Materials Research Laboratory, Key Laboratory of the Ministry of Education and International Center for Dielectric Research, Shaanxi Engineering Research Center of Advanced Energy Materials and Devices, School of Electronic Science and Engineering, Xi’an Jiaotong University, Xi’an, Shaanxi, China; 2 Lawrence Berkeley Natl Lab, Energy Storage and Distributed Resources Div, Berkeley, CA, United States

**Keywords:** novel materials and structures for photovoltaic technology, advanced nanomaterials for photocatalysts, advanced materials and structures for electrolytic hydrogen, advanced materials and structures for batteries and super capacitors, theoretical design for energy conversion and storage, solar desalination and water purification

## Introduction

The increasing global energy demand and the urgent need to mitigate climate change have stimulated an unprecedented surge of research into renewable energy conversion and storage technologies. Beyond incremental advances in mature systems such as silicon photovoltaics and lithium-ion batteries, researchers are now exploring disruptive materials and device architectures that can overcome fundamental efficiency limits, enable flexible or wearable configurations, and integrate energy harvesting with storage in a single platform. This Research Topic aims to highlight the most promising experimental and theoretical breakthroughs at the intersection of chemistry, materials science, and device engineering. The four contributions collected here span earth-abundant materials for solar cells and photocatalysis, solid-state electrolytes and redox-active frameworks for next-generation batteries and supercapacitors, as well as quantum dot-based infrared photodetectors. Collectively, these studies demonstrate how precise control over composition, morphology, crystal facets, surface chemistry, and interfacial coupling can be translated into higher device performance under realistic operating conditions.

## The contributing articles

In the first contribution, Xie et al. develop a low-temperature solvothermal route for producing highly crystalline SnO_2_ nanoparticles that can be directly dispersed in *n*-butanol and serve as efficient electron transport layers (ETLs) in perovskite solar cells. By varying the SnO_2_ concentration between 5 and 60 mg mL^-1^, the authors identify an optimal condition of 15 mg mL^-1^, which provides the best balance between film transparency, perovskite crystallinity, and interfacial charge recombination. The optimized devices achieve a power conversion efficiency (PCE) of 15.61% in rigid cells, while flexible counterparts fabricated on PEN/ITO substrates retain 94% of this performance (14.75%), which ranked among the highest PCE reported at that time for low-temperature flexible perovskite architectures. This work highlights that nanoscale engineering of ETLs can eliminate the high-temperature sintering step that has long restricted the roll-to-roll fabrication of lightweight and flexible photovoltaic modules.

In the second contribution, Bin et al. introduce a V_4_C_3_T_x_ MXene-reinforced polyvinyl alcohol (PVA) hydrogel electrolyte for flexible all solid-state supercapacitors. The electrolyte was fabricated via a cyclic freeze–thaw process, during which a three-dimensional MXene-bonded network was embedded within the PVA-H_2_SO_4_ matrix. The incorporation of MXene nanosheets significantly enhanced ionic conductivity from 105 to 133 mS cm^-1^ (PVA-H_2_SO_4_ vs. PVA-H_2_SO_4_-V_4_C_3_T_x_ MXene) and improved long-term cycling stability (99.4%@5500 cycles), while maintaining mechanical stretchability exceeding 200% strain. Symmetric supercapacitors using this optimized PVA-H_2_SO_4_-V_4_C_3_T_x_ MXene electrolyte achieved a capacitance of 370 F g^-1^ at 1 A g^-1^ and an energy density of 4.6 Wh·kg^-1^, nearly twice that of devices using pure PVA- H_2_SO_4_ electrolytes. This study exemplifies how two-dimensional carbides can synergistically enhance ion and electron transport in soft polymer systems, offering a scalable route toward deformable and self-healing power sources for next-generation wearable electronics.

In the third contribution, Dong et al. focus on electrocatalytic CO_2_ reduction to ethylene, an industrially important C_2_ feedstock. Cross-sectioned octahedral Cu_2_O microcrystals were prepared *in situ* on carbon paper electrodes by electrochemical deposition, where the morphology and exposure of the (111) crystal facet were precisely regulated by controlling the deposition potential, time, and temperature. During cathodic polarization, these well-defined Cu_2_O(111) surfaces reconstruct into metallic Cu^0^ nanosheets rich in low-coordination sites. The resulting catalyst achieves a Faradaic efficiency of 42% for C_2_H_4_ at −1.376 V vs. RHE in 0.1 M KHCO_3_ and maintains approximately 40% efficiency after 10 h of continuous electrolysis. Analyses confirm that the intact (111) facets of Cu_2_O serve as precursors for generating Cu^0^ domains that effectively promote *CO dimerization and C-C coupling, thereby offering a clear design principle for selective CO_2_-to-C_2_H_4_ conversion beyond the conventional Cu (100)-based paradigm.

Finally, in the fourth contribution, He et al. present an iodine-complex-directed synthesis (ICDS) strategy that enables the preparation of iodide-passivated PbS quantum dots (QDs) directly in polar solvents, thus avoiding lengthy hot-injection and ligand-exchange procedures. The dynamic balance between [PbI_3_]^-^ and [PbI_4_]^2-^ complexes ensure the controlled release of Pb^2+^ and I^−^ for *in-situ* surface passivation, while the absence of long-chain insulating ligands enhances interparticle coupling, producing a narrow photoluminescence emission at 1060 nm. When employed in sensitized photo-field-effect transistors, these PbS-I QDs deliver a specific detectivity of 1.63 × 10^11^ Jones and millisecond response times. In vertical photodiodes, they achieve rise and decay times of 10 and 15 µs, respectively, along with a detectivity of 1.12 × 10^11^ Jones under zero bias. This study demonstrates that ICDS at the synthesis stage can achieve electronic coupling and defect passivation comparable to those obtained by complex post-synthetic treatments. It demonstrates the potential of ICDS for enabling high-speed, low-noise NIR photodetectors.

We thank all authors for their insightful contributions and the reviewers for their constructive suggestions that sharpened each manuscript. Collectively, these studies illuminate a clear trajectory for the field: renewable energy devices will increasingly rely on chemically designed interfaces forged at low temperatures, self-assembled from earth-abundant elements, and capable of multifunctional operation under mechanical or environmental stress. We anticipate that the showcased concepts, including nanoscale engineering of ETL, MXene-hydrogel electrolytes, facet-controlled CO2 catalysts, and ligand-optimized QD inks, will seed translational research bridging laboratory records to pilot-line manufacturability, ultimately accelerating the global transition toward a resilient and carbon-neutral energy economy.

